# Optimization of the Microencapsulation Process of Açaí Extract (*Euterpe precatoria* Mart.) by Spray Drying and Its Effect on Bioactive Content, Solubility Capacity, and Hygroscopic Stability

**DOI:** 10.3390/foods15213914

**Published:** 2026-10-31

**Authors:** Fernando Castro-Armas, Braulio Bustamante-Oyague, Jessy Vásquez, Jorge Villacres-Vallejo, Daniel Guajardo-Flores, Daniela Guardado-Félix, Fernando Tello

**Affiliations:** 1 Departamento de Ingeniería de Alimentos, Facultad de Ingeniería Pesquera y de Alimentos, Universidad Nacional del Callao, Bellavista, Lima 07011, Peru; fernando.castro@unapiquitos.edu.pe (F.C.-A.); bbustamanteo@unac.edu.pe (B.B.-O.); 2 Departamento de Ingeniería de Alimentos, Facultad de Industrias Alimentarias, Universidad Nacional de la Amazonía Peruana, Caserío Zungarococha, San Juan Bautista, Iquitos 16002, Peru; jessy.vasquez@unapiquitos.edu.pe; 3 Departamento de Suelos y Cultivos, Facultad de Agronomía, Universidad Nacional de la Amazonía Peruana, Caserío Zungarococha, San Juan Bautista, Iquitos 16002, Peru; jorge.villacres@unapiquitos.edu.pe; 4 Escuela de Ingeniería y Ciencias, Tecnológico de Monterrey, Av. Eugenio Garza Sada 2501 Sur, Monterrey 64849, Nuevo León, Mexico; danielgdo@tec.mx; 5 Laboratorio de Nutracéuticos, Facultad de Ciencias Químico Biológicas, Universidad Autónoma de Sinaloa, Culiacán 80010, Sinaloa, Mexico; daniela.guardado@uas.edu.mx

**Keywords:** Amazon fruits, açai extract, bioactive compounds, antioxidant capacity, microencapsulation, spray drying, response surface methodology

## Abstract

*Euterpe precatoria* Mart. (açaí) is a superfruit with high nutritional and functional value; however, its high perishability accelerates the degradation of its bioactive compounds. Therefore, technologies such as microencapsulation allow for its preservation, improve its bioavailability, and extend its shelf life. The objective of this study was to optimize the microencapsulation process of açaí extract via spray drying, using response surface methodology (RSM) and a central composite design (CCD), in order to evaluate the effect of inlet air temperature (IAT) and the gum Arabic: maltodextrin (GA:MD) ratio on the total phenolic content (TPC) and total anthocyanin content (TAC), antioxidant capacity (ABTS, DPPH), as well as on the water-solubility index (WSI) and hygroscopicity (HYG) of the resulting powder. The desirability function (D) indicated that an IAT of 135 °C and a GA:MD ratio of 25:75 constitute the optimal conditions for spray-drying the açaí extract, under which the following values were obtained: 93.49% WSI, 7.42% HYG, 3291.22 mg GAE/100 g TPC, and 115.89 mg C3G/100 g TAC. Experimental validation yielded values within the 95% confidence interval established for the model’s predictions. Scanning electron microscopy analysis (SEM) revealed predominantly spherical açaí powders with smooth or slightly wrinkled surfaces, and microparticle diameters ranging from 2.10 to 24.22 μm. This indicates that, under the conditions evaluated, favorable results were obtained in terms of the physicochemical properties and the retention of TPC (up to 92.15%) and TAC (up to 84.60%) in the powder. Therefore, these results provide an experimental basis for the development of value-added products from Amazonian fruits.

## 1. Introduction

Plant-based foods have gained increasing importance in human nutrition, which is why their consumption has been widely promoted as part of healthy dietary patterns [1], with a recommended minimum intake of 400 g per day [2]. In this context, Amazonian fruits represent an exceptional source of bioactive compounds of great importance for human health and the food industry [3]. In this context, the fruit of *Euterpe precatoria* Mart. (huasaí or Amazonian açaí), a palm tree of the Arecaceae family native to the Amazon, has emerged as a resource of exceptional nutritional and functional value [4]. From a compositional perspective, this species stands out for its remarkable dietary fiber (27.31%), lipids (5.75%), and protein (12.73%) content [5]. However, its relevance in the functional food industry lies primarily in its rich phytochemical profile. Studies focused on characterizing its extracts have reported concentrations of phenolic compounds of up to 3964,32 mg GAE/100 g, with a total anthocyanin content ranging from 128.4 to 868.9 mg/100 g [6,7], where the anthocyanin forms cyanidin-3-O-rutinose and cyanidin-3-O-glucoside are the predominant ones in the group of these water-soluble flavonoids responsible for the fruit’s characteristic purple pigmentation [8,9].

In this regard, these anthocyanins have been widely documented for their ability to reduce the risk of cardiovascular disease, neurological disorders, type 2 diabetes, and various types of cancer, thanks to their potent cardioprotective, antioxidant, and anti-inflammatory properties [1,10]. However, despite this nutraceutical and functional potential, the agroindustrial utilization of *Euterpe precatoria* faces critical challenges during processing, storage, and distribution due to its high perishability and the marked instability of the anthocyanins, as these compounds are highly sensitive to extrinsic and intrinsic factors such as pH, oxygen, temperature, light radiation and water activity [11,12], which constitute the main obstacle to its application as colorants and nutritional and functional ingredients in the food industry.

Given this scenario, technological dehydration processes using concentrated extracts represent a viable option for recovering and preserving the structural integrity of these bioactive compounds. Microencapsulation via spray drying has established itself as the most efficient and scalable technology for protecting heat-labile bioactive compounds, improving their physicochemical stability and facilitating their incorporation into various food matrices within the food industry [13,14]. This process involves the atomization or nebulization of a solution containing the bioactive material and the encapsulating material, followed by the rapid evaporation of the solvent within a drying chamber, ultimately resulting in powder-like microparticles [15].

Conventional encapsulation materials include maltodextrin (MD), and gum Arabic (GA), which are notable for their film-forming properties, their ability to generate protective matrices against degradation factors, and their compatibility with bioactive compounds such as phenolics [16]. Studies on the spray-drying of species of the genus *Euterpe* have reported encapsulation efficiencies ranging from 80.27% to 99.50% using biopolymers and anthocyanin retention rates of 55% to 95%, depending on process conditions [17], while GA:MD combinations have demonstrated superior results in powder stability and bioactive retention in various plant matrices. Tan et al. [18] reported a yield (PY) of 66.2% and retention rates exceeding 79.5% in aqueous bitter melon extract. Similarly, Anisuzzaman et al. [19] reported the lowest hygroscopicity (HYG: 5.67%) in tomato powder produced using these parameters and technology. Likewise, Sarabandi et al. [20] reported a water-solubility index (WSI) greater than (94.31%) in eggplant extract, demonstrating the suitability of this biopolymer blend for spray drying.

In this context, the physicochemical properties of the resulting powder are strongly influenced by two main process variables: inlet air temperature (IAT) and the concentration ratio of the encapsulating materials [21,22]. IAT determines the rate of water evaporation, the formation of protective membranes, and the retention of heat-labile bioactive compounds, as moderate IAT values (120 to 160 °C) have been shown to result in higher anthocyanin retention compared to extreme conditions [23,24]. In turn, the concentration of the encapsulating materials alters the viscosity of the feed solution and influences the powder size, HYG, and WSI of the final product [25,26]. A parameter of particular relevance to the stability of these powders is the glass transition temperature (Tg), which represents the transition of the amorphous matrix from a glassy solid to a rubbery or liquid state [27]. In fruit extracts, which typically contain high concentrations of low-molecular-weight sugars and organic acids, the Tg can decrease significantly, leading to issues such as stickiness, structural collapse, and premature release of microencapsulated compounds during processing and storage [28,29]. Therefore, it is essential to understand and control this parameter (Tg) as part of the design of spray-drying processes in order to ensure the long-term stability of the powder.

The simultaneous optimization of these variables using response surface methodology (RSM) provides an efficient mathematical and statistical framework that reduces the number of experiments required and allows for the modeling of multiple responses [30]. Spray drying of açaí has been studied over the past few decades. Previous studies, such as those by Tonon et al. [31], Oliveira et al. [32], Valente et al. [33], Silva et al. [34], and Vargas et al. [35], focused on *Euterpe oleracea* and *edulis* as raw materials, using MD as the encapsulating material. More recently, Tello et al. [7] optimized the spray-drying of *Euterpe precatoria* extract using GA:MD and established optimal conditions for PY, HYG, and TPC; however, WSI and TAC could not be optimized due to a lack of significant model fit. Consequently, despite advances in the microencapsulation of *Euterpe* species, there is still limited knowledge regarding the multi-response optimization of spray-drying *Euterpe precatoria* extracts, a species of biological and economic importance in the Amazonian regions of Peru, Colombia, and Brazil [36]. In this context, given the differences in nutritional and functional profiles among species, and the need to develop technologies adapted to Amazonian resources with the potential to create regional value, it is essential to investigate the optimal drying conditions for extracts of this species, especially when the physicochemical and bioactive properties of the final product are considered simultaneously.

Therefore, the objective of our study was to optimize the microencapsulation process of açaí extract (*Euterpe precatoria* Mart.) using spray drying and to evaluate the effect of IAT and the GA:MD ratio at 10% total solids on the TPC and TAC, antioxidant capacity (ABTS, DPPH), WSI, and HYG of the resulting powder. Thus, this approach is expected to contribute to the development of functional products for use in functional foods on a global scale.

## 2. Materials and Methods

### 2.1. Materials and Reagents

Fresh açaí fruits of the variety *Euterpe precatoria* Mart. used in this study were obtained from the rural settlement of Huambé (km 50), located in the city of Iquitos in the province of Maynas, Loreto region, Peru. The encapsulation materials were MD (DE 10) and GA, purchased from Sigma-Aldrich^®^, St. Louis, MO, USA. Likewise, the reagents used, including Folin–Ciocalteu reagent, Trolox as a standard, KCl, HCl, and CH_3_COONa, as well as Milli-Q water and ethanol (99.8%, analytical grade), were purchased from Sigma-Aldrich^®^, St. Louis, MO, USA, and Merck^®^, Darmstadt, Germany, respectively. In general, all other chemicals used in the present study were analytical-grade and were acquired from recognized suppliers.

### 2.2. Preparation of the Extract

The extraction of bioactive compounds was carried out following the methodology proposed by Pompeu et al. [37], with some modifications. The acai pulp, obtained by first soaking the fruits in water at 55 °C for 45 min and then processing them in a vertical pulper equipped with a 0.6 mm screen (Metvisa, model DG20, Brusque, Brazil), was stored in HDPE containers at −18 °C until use. The pulp was then macerated in a hydroalcoholic solution of ethanol and water (70:30, *v*/*v*). A solid-to-solvent ratio of 1:2.5 (*w*/*v*) was used, corresponding to 1 kg of pulp per 2.5 L of extracting solution. The mixture was kept protected from light for 72 h at room temperature (31 ± 1 °C). Subsequently, the extract was filtered using Whatman No. 1 filter paper and concentrated in a rotary evaporator (MRC Laboratory Instruments, model ROVA-100, Holon, Israel) at 50 °C and 120 mbar for 20 min. The concentrated extract had a total solids content of 30% and a residual alcohol content of 9%. Table 1 shows the phenolic compound content of the pulp and hydroalcoholic extract of açai.

### 2.3. Production of Microencapsulated Powder by Spray Drying

The microencapsulated powder was produced by spray drying, following the methodology proposed by Ferreira et al. [38] with some modifications. A feed solution with a total solids content of 10% (*w*/*w*) was prepared, in which the solid fraction consisted of 30% concentrated extract and 70% encapsulating material, calculated based on the total solids in the formulation (feed solution). The solution was homogenized at room temperature (31 ± 1 °C) under mechanical agitation at 400 rpm for 10 h, at which point 1000 mL of solution was pumped into the drying chamber for each run using a peristaltic pump. Spray drying was performed in a laboratory-scale spray dryer (model SD-05, LabPlant UK Ltd., Filey, UK), equipped with a spray nozzle with an internal diameter of 1.5 mm and a drying chamber measuring 500 × 215 mm. Operating conditions included a dry air flow rate of 73 m^3^/h, a spray pressure of 0.06 MPa, and a feed rate of 10 mL/min. The outlet air temperature ranged from 67 to 84 °C. The experimental tests were conducted using a CCD, with IAT and GA:MD ratio as independent variables. After drying, the microencapsulated powders were immediately collected in airtight glass jars, protected from light, and stored at −18 °C until analysis.

### 2.4. Process Yield

The PY of the microencapsulation process was determined using the methodology proposed by Chang et al. [39], in which quantification was based on the ratio of the mass of the powder recovered after spray drying to the total mass of solids in the feed solution, expressed as a percentage according to Equation (1):

(1) $$PY\;\left(\%\right)=\frac{Collected\;spray\;dried\;powder\;(g)}{Total\;solids\;in\;the\;feed\;solution\;(g)\;}\;\times \;100$$

### 2.5. Water-Solubility Index

The WSI of the powder was determined using the methodology proposed by Pieczykolan and Kurek [40], with some modifications. First, 1 g of powder was dispersed in 100 mL of distilled water by stirring for 5 min. The resulting suspension was then centrifuged at 3000× *g* for 15 min in a Hettich Model 320R centrifuge, Tuttlingen, Germany. Subsequently, 25 mL of the supernatant was transferred to a previously tared beaker and dried in an oven (Thermo Scientific^®^, Heratherm^TM^ model, Langenselbold, Germany) at 105 ± 1 °C for 24 h until a constant weight was reached. WSI was calculated in relation to the dissolved solids, expressed as a percentage according to Equation (2):

(2) $$WSI\;\left(\%\right)=\frac{Recovered\;soluble\;solids\;(g)}{0.25}\;\times \;100$$

### 2.6. Moisture

The MC of the powder was determined using the method established by the AOAC [41]. Approximately 5 g of powder was weighed into Petri dishes and then dried in an oven (Thermo Scientific^®^, Heratherm^TM^ model, Langenselbold, Germany) at 105 ± 1 °C for 6 h. The MC was quantified by weight difference, according to Equation (3):

(3) $$MC\;\left(\%\right)=\frac{Initial\;sample\;weight\;\left(g\right)-final\;sample\;weight\;after\;drying\;(g)}{Initial\;sample\;weight\;(g)}\;\times \;100$$

### 2.7. Hygroscopicity

The HYG of the powder was determined using the methodology proposed by Cai and Corke [42], with some modifications. Approximately 2 g of powder was placed in Petri dishes and placed in a desiccator maintained at an RH of 75% and a temperature of 28 ± 1 °C using a saturated NaCl solution. The samples were kept under these conditions for 7 days. HYG was quantified based on the increase in powder mass following exposure to the controlled environment, expressed as a percentage according to Equation (4):

(4) $$HYG\;\left(\%\right)=\frac{Water\;absorbed\;by\;the\;powder\;(g)}{Initial\;powder\;mass\;(g)}\;\times 100$$

### 2.8. Extraction of Bioactive Compounds from the Powder

The extraction of bioactive compounds from the powder was performed following the methodology proposed by Rufino et al. [43], with some modifications. Approximately 1 g of powder was weighed into a Falcon tube and mixed with 5 mL of a hydroalcoholic ethanol: water solution (1:1, *v*/*v*) and allowed to stand for 1 h at 31 ± 1 °C in the dark. Subsequently, the suspension was centrifuged at 3000× *g* for 15 min at 25 ± 1 °C. The supernatant was collected and filtered using Whatman No. 1 filter paper. The residue was subjected to a second extraction with 10 mL of the same hydroalcoholic solution under the same conditions; the two extracts were then combined to yield a final extract volume of 15 mL and then analyzed.

### 2.9. Total Phenolic Content

The TPC of the powder was determined using the Folin–Ciocalteu method proposed by Singleton et al. [44], with some modifications. Firstly, 20 μL of the extract obtained from the powder was diluted in 9980 μL of ethanol, yielding a 1:500 dilution, which was homogenized. Next, 200 μL of this solution was taken and mixed with 1500 μL of distilled water and 100 μL of Folin–Ciocalteu reagent, and the mixture was allowed to react for 5 min at room temperature (31 ± 1 °C). Next, 200 μL of a 20% (*w*/*v*) Na_2_CO_3_ solution was added. The mixture was incubated in the dark for 30 min, and the absorbance was measured at 765 nm using a UV-Vis spectrophotometer (Thermo Scientific^®^, Waltham, MA, USA; model GENESYS 150). TPC was quantified using a calibration curve prepared with gallic acid as the reference standard. The results were expressed in mg of GAE/100 g of extract, calculated according to Equation (5):

(5) $$TPC\;\left(mg\frac{GAE}{100}\;g\right)=\frac{Concentration\;GAE\;(mg/L)\;\times \;dilution\;factor\;\times \;volume\;of\;extract\;\left(L\right)\;\times \;100\;}{mass\;of\;extract\;(g)\;}$$

### 2.10. Total Anthocyanin Content

The TAC of the powder was determined using the differential pH method proposed by Lee et al. [45], with some modifications. In 10 mL volumetric flasks, 200 μL of the extract obtained from the powder was diluted in 9800 μL of buffer solutions consisting of KCl (0.025 M) at pH 1.0 and CH_3_COONa (0.4 M) at pH 4.5. The combined solutions were then allowed to stand for 15 min at room temperature (31 ± 1 °C), in the absence of light. The absorbances measured during the sweep at 520 and 700 nm were recorded using a UV-Vis spectrophotometer (Thermo Scientific^®^, Waltham, MA, USA; model GENESYS 150). The TAC was calculated according to Equations (6) and (7) and expressed as mg C3G/100 g of extract, where the constants used were the molecular weight of cyanidin-3-glucoside (449.2 g/mol); the molar absorptivity coefficient of cyanidin-3-glucoside (26,900 L mol^−1^·cm^−1^); and the cell path length (1 cm). Furthermore, the dilution factor was that of the mixture of the combined extract and the buffer solutions; the mass of the extract was the amount of extract used in the analysis and 100 was used as the quantity to convert mg of C3G per 100 g of extract.

(6) $$Acorrected=\left(A520\;nm-A700\;nm\right)pH\;1.0-(A520\;nm-A700\;nm)pH\;4.5$$

(7) $$TAC\;\left(mg\frac{C3G}{100}g\right)=\frac{Acorrected\times molecular\;weight\;(g/mol)\times dilution\;factor\times volume\;of\;extract\;(ml)\times 100\;}{Molar\;coefficient\;(l/mol\cdot cm)\times cell\;length\;\left(cm\right)\times mass\;of\;extract\;(g)\;}$$

### 2.11. Antioxidant Capacity

#### 2.11.1. ABTS Method

The antioxidant capacity of the powder, as determined by the ABTS•^+^ assay, was measured according to the methodology proposed by Re et al. [46], with some modifications. The ABTS•^+^ radical was generated by the reaction between an aqueous solution of ABTS (7 mM) and K_2_S_2_O_8_ (2.45 mM). The mixture was incubated in the dark for 12 to 16 h at room temperature (31 ± 1 °C). Subsequently, the ABTS•^+^ solution was diluted with ethanol until an absorbance of 0.70 was obtained at 734 nm. To determine the antioxidant capacity, 30 μL of the extract obtained from the powder was mixed with 3000 μL of the ABTS•^+^ solution. The mixture was allowed to react for 6 min at room temperature (31 ± 1 °C) in the dark. Finally, the absorbance was measured at 734 nm using a UV-Vis spectrophotometer (GENESYS 150, Thermo Scientific^®^, Madison, WI, USA).

#### 2.11.2. DPPH Method

The antioxidant capacity of the powder, as determined by the DPPH• assay, was measured according to the methodology proposed by Brand-Williams [47], with some modifications. Firstly, 3900 μL of an ethanolic DPPH• solution (0.1 mM) was mixed with 100 μL of the extract obtained from the powder. The mixture was then incubated in the dark for 30 min at room temperature (31 ± 1 °C). Subsequently, the absorbance was measured at 517 nm using the same UV-Vis spectrophotometer used for the ABTS assay. In both assays, antioxidant capacity was quantified using calibration curves constructed with Trolox as the reference standard. The results were expressed μmol ET/g, according to Equation (8):

(8) $$ABTS;DPPH\;\left(µmol\;ET/g\right)=\frac{Concentration\;(µmol/L)\times \;volume\;of\;extract\;\left(L\right)\times \;dilution\;factor\;}{mass\;of\;extract\;(g)}$$

### 2.12. Morphological Properties

The morphology of the powder was characterized using the methodology proposed by Ibrahim et al. [48]. The powder was analyzed by scanning electron microscopy (SEM) using a Nova NanoSEM 200 (FEI, Hillsboro, OR, USA). The powder was mounted on a metal substrate using carbon adhesive tape and coated by sputtering with a thin conductive layer of gold (model FDU-010, Balzers, Liechtenstein) to obtain micrographs under vacuum conditions, using acceleration voltages between 5 and 15 kV and magnifications of 1000× and 2500×. To determine the diameter and polydispersity index (PDI), the powder was suspended in 15 mL of Milli-Q water to a concentration of 30 mg/mL and homogenized using a vortex mixer; subsequently, 6 mL aliquots were transferred to cuvettes for analysis by dynamic light scattering (DLS) using a Zetasizer Nano (Malvern Instruments, Malvern, UK) at 25 °C, with the absorbance set to 0.8 and the refractive index set to 1.475. Finally, to determine the zeta potential (ZP), 1 mL of the same suspension was transferred to a folded capillary cuvette and analyzed by electrophoretic light scattering using the same instrument.

### 2.13. Data Analysis

The statistical analysis was performed using Statistica^®^ v. 12.0 software (StatSoft Inc., Tulsa, OK, USA). The experimental data obtained from the CCD, consisting of 13 experimental entries (2^2^ factorial points, 2 k axial points, and 5 central points) performed in 3 separate spray-drying runs, were fitted to a second-order response surface model using multiple polynomial regression, where the response variables were PY, WSI, HYG, TPC, TAC, and ABTS and DPPH levels. The normality of the residuals for each fitted model was assessed using the Shapiro–Wilk test, where the model’s goodness of fit was evaluated via ANOVA, considering the statistical significance of the model terms (*p* < 0.05), R^2^, and lack of fit. Optimization of the response variables was performed using MSR, and the global desirability function (D) was employed to identify the optimal operating conditions. Finally, the predictive model was validated through confirmatory experiments conducted in triplicate, in which the relative deviation was calculated as follows:

(9) $$Relative\;deviation\;(\%)=(\frac{Predicted\;value-Observed\;value\;}{Observed\;value\;})\;\times \;100$$

## 3. Results and Discussion

### 3.1. Optimization of Powder Production

The CCD, which includes the coded and decoded levels of the independent variables, along with the corresponding experimental response variables, is presented in Table 2. This design was employed within the RSM framework to investigate the effects of the process variables; thus, this multivariate approach allowed the simultaneous optimization of multiple quality attributes of the microencapsulated powder [49].

The ANOVA results for the response variables are presented in Table 3. The models for PY, MC, ABTS, and DPPH were excluded from the analysis due to a significant lack-of-fit test (*p* < 0.05), indicating that the proposed polynomial model failed to adequately represent the variability of the experimental data, possibly due to higher-order effects not captured by the quadratic model or to the low pure error observed in the midpoint replicates, which increased the test’s sensitivity. IAT and the GA:MD ratio showed significant linear effects on WSI, HYG, TPC, and TAC. Both factors also exhibited significant quadratic effects on WSI, TPC, and TAC. In contrast, for HYG, only the quadratic effect of the GA:MD ratio was significant, not that of IAT. No significant interactions were detected between the two factors. Although the linear effect of the IAT showed greater statistical evidence than that of the GA:MD ratio for all four response variables, this does not necessarily imply an effect of greater magnitude. The regression analysis obtained by fitting second-order polynomial models allowed the description and optimization of the response variables, according to the following equations:

% WSI = 52.34856 + 0.52648X_1_ − 0.00165X_1_^2^ − 0.41582X_2_ − 2.65704X_2_^2^ + 0.01592X_1_X_2_

% HYG = −5.5664497 + 0.0600175X_1_ + 0.0002263X_1_^2^ + 6.2029990X_2_ − 2.3463807X_2_^2^ − 0.0170991X_1_X_2_

mg GAE/100 g TPC = −18,502.24934+343.40537X_1_ − 1.36148X_1_^2^ + 1023.16615X_2_ − 1356.82692X_2_^2^ + 3.64387X_1_X_2_

mg C3G/100 g TAC = −347.882101 + 7.238863X_1_ − 0.028291X_1_^2^+40.623471X_2_ − 18.914579X_2_^2^ − 0.196934X_1_X_2_

#### 3.1.1. Powder Yield

The PY is a key parameter in industrial settings because of its close relationship to process efficiency and production costs [50]. In this study, PY ranged from 47.96% to 53.82%, reaching its maximum value in experiment 6, at a high IAT (150 °C), followed by experiment 13, at a moderate IAT (135 °C), both at the same biopolymer ratio (GA:MD 50:50), while the minimum value was observed in experiment 2, at a low IAT (124.4 °C) and a higher proportion of GA (GA:MD 90:10.), indicating that both the IAT and the biopolymer ratio (GA:MD) significantly influenced powder recovery.

The results obtained are consistent with those described by Tonon et al. [51] and Valente et al. [33], who reported a PY ranging from 34.39 to 55.94% for açaí powders produced using MD. The properties of the encapsulants could contribute to the differences in PY, as microparticle adhesiveness has been linked to surface temperatures higher than the matrix’s Tg [52]. Possible contributions of MD to the formation of glassy matrices and of GA to interfacial stabilization have been described [53]. In this regard, Tello et al. [7] reported a PY of up to 70.75% for açaí powders produced at an IAT of 125 °C and a GA:MD ratio of 50:50; however, they obtained up to 69.45% at 142 °C and a GA:MD ratio of 90:10. These differences could be related to processing conditions, including the solids content in the feed, which was 8% compared to the 10% used in this study; and although the loss was not quantified, dust retention was observed primarily in the equipment’s cyclone. Goula and Adamopoulos [54] linked the decrease in PY at high MD concentrations to increases in viscosity, the size of the atomized microdroplets, and deposition on the dryer walls.

The minimum PY observed in Experiment 2 is attributed to the system’s low internal IAT (124.4 °C), likely because it limits the kinetics of water evaporation and prolongs the residence time of the microparticle in the rubbery state. Tan et al. [55] and Jafari et al. [56] note that a high IAT (124 to 143 °C) promotes faster surface evaporation, which forms a solid crust on the microparticle before it impacts the chamber walls, thereby reducing adhesion and increasing the PY—a behavior consistent with that reported by Decker et al. [57], in the atomization of grape pomace (PY: 43.02 to 50.00%). However, Tay et al. [58] note that excessive IAT thermal intensity can cause surface softening or partial collapse of the microdroplets, thereby reducing the process PY; this was corroborated by Gawałek [59] in Aronia juice powder when using IAT above 170 °C (PY: 93 to 83%). Therefore, the identified combination strikes a balance between the formation of a rigid, amorphous matrix and the interfacial protection it provides, maximizing powder recovery; despite the lack of fine-tuning in the optimization, according to Bhandari et al. [60], this is considered successful because it achieves PY values greater than 50%.

#### 3.1.2. Water-Solubility Index

The WSI is a critical quality parameter that determines the rehydration capacity of powders and their suitability for use in food formulations [61]. In the present study, the açaí extract powders exhibited solubility values ranging from 91.36% to 94.31%, with the maximum value recorded in experiment 6 at a high IAT (150 °C) and a biopolymer ratio (GA:MD; 0.5:0.5), followed by experiment 3 at a slightly high IAT (145.6 °C), with a higher proportion of MD (GA:MD; 10:90), while the minimum value was recorded in experiment 2 at a low IAT (124.4 °C) with a higher proportion of GA (GA:MD; 90:10), suggesting that a moderately high IAT and higher proportions of MD favor the WSI of the powders.

In our study, the effect of the GA:MD ratio on the WSI was significant (*p* < 0.05); in particular, when pure MD or a higher proportion of MD was used, a high WSI was observed. These results are consistent with those reported by Rocha et al. [62] for Jabuticaba, Jussara, and blueberry powders; by using more MD than GA and Whey protein concentrate, these researchers achieved a WSI of up to 90.27%. These results could be attributed, on the one hand, to the prior filtration of the extracts, which likely reduced the presence of suspended microparticles and insoluble material; and, on the other hand, to the DE and the high WSI of the MD due to the presence of hydroxyl groups (-OH) capable of interacting with water during reconstitution [63,64]. In contrast, in our study, the formulations with a higher proportion of GA showed lower WSI, likely due to their less porous structure composed of arabinogalactan and protein fractions, which slows their dissolution in water [65], as noted by Asik et al. [66] in myrtle powder, reporting a WSI of 41.29%, and Santana et al. [67] in Jussara powder (77.99%). This could explain the synergistic effect of biopolymers in stabilizing and improving the WSI, which is comparable to the findings of Estupiñán-Amaya et al. [68], who reported WSI values exceeding 94% in blueberry powder.

The influence of IAT on the reconstitution of powders in aqueous media has been reported. In our study, the effect of IAT on the WSI of the powder was significant (*p* < 0.05), with an effect comparable to that described by Hadree et al. [69] for pomegranate juice powder and by Silva Júnior et al. [70] in ciriguela powder; when they used an IAT ranging from 160 to 190 °C and 130 to 170 °C, they recorded a WSI of 89.5 to 91.94% and 86.43 to 92.20%, respectively. This effect is likely due to the lower moisture content of the developed powders, which favored the formation of a more vitreous, rough, and porous structure, thereby facilitating water penetration and preventing agglomeration during reconstitution [71]. Therefore, in our study, we did not observe a significant decrease in WSI within the evaluated range, suggesting that the IAT was suitable for the formation of dispersible and rehydratable matrices without causing excessive surface crusting.

#### 3.1.3. Moisture Content and Hygroscopicity

MC is a critical parameter in atomized powders, as it determines their physicochemical stability and flowability [72]. In our study, the MC ranged from 2.01 to 4.06%. In our study, we observed that an increase in the IAT caused it to decrease progressively, which was associated with an increase in the rates of heat and mass transfer promotes extremely rapid evaporation of water on the surface of the atomized droplets [73]. Similar results were reported by Qadri et al. [28] in apple powder (MC: 6.71 to 2.18%). Likewise, powders containing GA had higher MC, likely due to their branched structure and polar groups [74], reaching the lowest levels when a 50:50 ratio of GA:MD was used. The Tg of the encapsulating plays a critical role in this process; water acts as a plasticizer, reducing the matrix’s Tg and promoting the transition from the glassy state to the rubbery state, which dramatically increases molecular mobility and water-adsorption capacity [75].

HYG is a critical physicochemical property for dry powders; it determines the material’s intrinsic ability to absorb water vapor from the environment, a factor that influences its stability and shelf life during storage [76]. In our study, the HYG ranged from 5.62 to 10.05%, with the lowest value recorded in experiment 1 at a low IAT (124.4 °C), with a higher proportion of MD (GA:MD; 10:90), while the highest value was observed in experiment 6, at a high IAT (150 °C) and an equal ratio of biopolymers (GA:MD; 50:50), followed by experiment 4, in which a slightly higher IAT (145.6 °C) and a higher proportion of GA (GA:MD; 90:10) were used. The results obtained are comparable to those described by Valente et al. [33] for açaí powder using MD (HYG: 7.87%). According to Vidović et al. [77], the effect of MD on HYG depends on its DE and mass fraction, showing that a higher proportion of MD and a low DE were associated with a lower HYG (12.4%), likely due to the long glucose chains that form compact structures with low -OH groups, which reduces moisture affinity [78,79]. In our study, we observed that the powder containing GA exhibited higher HYG, a finding that was corroborated by Ijod et al. [80] in black grape powder (HYG: 19.80%), due to its open, highly branched three-dimensional structure with a high density of polar groups (-OH, -COOH, and -NH_2_) that act as sites for water adsorption via hydrogen bonds [81].

Furthermore, the synergy of the encapsulant mixture (50:50) at a moderate IAT appears to stabilize the HYG of the powder in our study; these results are consistent with those reported by Tello et al. [7] for açaí powder (HYG: 7.12 to 7.58%). According to Bazaria and Kumar [82], this effect is due to the fact that the MD acts as a diluent for the hydrophilic groups of the GA, a process that is reversed when the proportion of GA exceeds a critical threshold, highlighting the influence of its hydrophilic nature. However, in our study, this behavior was more pronounced with IAT, and the difference was statistically significant (*p* < 0.05). Comparable results were reported by Lima et al. [83] for jucara powder and by Vargas et al. [84] for açaí and copoazú powders; when using an IAT from 110 to 180 °C, they reported an HYG from 11.08 to 13.91%. Furthermore, these authors attributed this behavior to the powders’ low moisture content, associated with the rapid evaporation of water during drying. Therefore, the HYG results indicated that the açaí powders exhibited low water absorption under the specific 7-day conditions evaluated, a characteristic that could promote their physicochemical stability during storage.

#### 3.1.4. Total Phenols

The retention of TPC during spray drying represents one of the technological challenges in the microencapsulation of bioactive compounds, as they are thermolabile and susceptible to degradation [85]. In our study, TPC values ranged from 2733.32 to 3618.66 mg GAE/100 g, with the highest value recorded in experiment 5 at a low IAT (120 °C), followed by experiment 11 at a moderate IAT (135 °C), while the minimum was observed in experiment 6 at a high IAT (150 °C), all with a proportional GA:MD ratio (50:50). IAT was the predominant variable that significantly influenced (*p* < 0.05) TPC. After spray drying, retention—expressed as equivalent dry extract—ranged from 69.61 to 92.15%.

During drying, thermolabile phenols undergo bond breaking, degradation, isomerization, and oxidation due to high temperatures, oxygen, and reduced water activity [86]. As the microparticle crust forms, the phenols near the surface are exposed to greater thermal gradients and increased oxygen diffusion, which promotes their degradation or polymerization into structures with higher molecular weights [87,88]. The behavior observed in our study is comparable to that described by Gawałek et al. [88], in *Aronia melanocarpa* powder; they observed a systematic decrease in TPC (from 3217 to 1965 mg GAE/100 g). Vidović et al. [89] noted that an IAT above 140 °C accelerates the degradation of TPC in aronia powder (from 325.1 to 178.5 mg GAE/g). In contrast, Can et al. [90] reported that at an IAT of 120 °C, a higher concentration of TPC (87.12%) is retained in blueberry extract powder. However, the effect of IAT on TPC is not consistent across all plant matrices. Vargas et al. [84] observed that an increase in the drying temperature raised the TPC in Cupuassu and açaí powders (from 943.85 to 2089.88 mg/100 g), due to the brief residence time of the microparticles in the drying chamber. This phenomenon can also be attributed, in certain polyphenols, to the hydrolysis of phenolic–sugar bonds and the formation of more reactive aglycones [44,91]. In our study, this trend was not observed, especially at an IAT of 150 °C.

The effect of IAT can be modulated by using biopolymers as encapsulation materials. In our study, the GA:MD ratio also significantly influenced (*p* < 0.05) the TPC, although GA alone showed a greater ability to preserve the TPC compared to MD. This behavior is consistent with that described by Silva Júnior et al. [92] in plum extract powder (451.2 mg GAE/g). In our study, the AG:MD mixture (50:50) had a greater effect on TPC; these results are consistent with those of Cid-Ortega and Guerrero-Beltrán [93] for *Hibiscus sabdariffa* powder (3801.6 mg GAE/100 g). It is also consistent with the findings of Tello et al. [7] for açaí powder, although their values (2202.45 to 2803.29 mg GAE/100 g) were lower than those observed in our study. These differences can be attributed to the total solids concentration; according to Correâ-Filho et al. [94], a higher concentration increases the viscosity of the feed solution, which limits the mobility and surface migration of TPC within the droplet, thereby reducing its direct exposure to heat and oxygen. Therefore, the synergistic effect of both biopolymers can be explained by the fact that MD, depending on its DE, has the ability to form amorphous glassy matrices around the core. Meanwhile, GA exhibits a greater affinity for phenolic molecules due to its hydroxyl groups and protein regions, which are capable of establishing intermolecular interactions via hydrogen bonds and electrostatic forces, leading to the formation of continuous and resistant films [95,96].

#### 3.1.5. Total Anthocyanins

The same trend observed for TPC was also evident for TAC, where the effect of IAT was highly significant (*p* < 0.05). TAC values ranged from 101.36 to 118.57 mg C3G/100 g, with the maximum recorded in experiment 5 at low IAT (120 °C), followed by experiment 13 at moderate IAT (135 °C), and the minimum obtained in experiment 6 at high IAT (150 °C), all with a proportional GA:MD ratio (50:50). These results suggest that the IAT and the GA:MD ratio exert a combined influence on the retention of bioactive compounds, as reflected in the coincidence of the maximum TPC and TAC values. TAC retention values, expressed in terms of the equivalent dry extract, ranged from 72.32% to 84.60% following the spray-drying process.

Anthocyanins are susceptible to degradation by temperature, oxygen, and light due to the intrinsic instability of the flavylium cation [97]. Dong et al. [98] demonstrated that TAC degradation occurs through cleavage of the glycosidic bond and release of cyanidin and glucose, followed by heterocyclic ring opening and the formation of chalcone-like structures, and finally degradation into low-molecular-weight compounds. Furthermore, degradation has been shown to be almost complete in an air atmosphere [12]. In spray drying, high IAT generally cause powders to collapse and crack, which paradoxically allows greater oxygen ingress and greater thermal exposure of the interior [99]. In our study, a progressive reduction in TAC was observed, especially when the IAT exceeded 145.6 °C. Nguyen et al. [100] noted that increasing the IAT (to 150, 160, and 170 °C) significantly affected the TAC in *Hibiscus sabdariffa* powder (from 10.47 to 6.08 mg of C3G/g), due to oxidation, hydrolysis, and structural transformation of the flavyl cation. Furthermore, studies such as those by Tonon et al. [51] and Vargas et al. [35] confirmed this effect when the researchers produced açaí powder at IAT of 110 and 202 °C (up to 162.40 mg of C3G/100 g). Consequently, the lower TAC values observed at 150 °C confirm the high thermal instability of these pigments during spray drying.

On the other hand, the effect of the GA:MD ratio was also significant (*p* < 0.05), with formulations containing only MD retaining a higher concentration of TAC. In this regard, Rigolon et al. [101] reported TAC levels of up to 111.0 mg/100 g in açaí powder using MD, compared to 99.99 mg/100 g with GA. However, in our study, the formulations with an equimolar ratio of GA:MD presented the highest TAC values. This finding is consistent with that described by Pauletto et al. [102] for jaboticaba powder and Tello et al. [7] for açaí powder, as these authors reported concentrations ranging from 72.12 to 155.79 mg of C3G/100 g of TAC. This advantage of MD can be explained by its lower viscosity in solution, which allows for higher concentrations of soluble solids and causes the system to form a continuous, glassy outer crust as well as spherical microparticles with smooth surfaces—conditions that would reduce the pathways through which hot air and oxygen reach the TAC [103,104]. Although GA exhibits excellent film-forming ability, its composition leads to the formation of wrinkled microparticles that tend to collapse and exhibit a higher HYG, resulting in regions of molecular mobility where the TAC would be more exposed to oxidation [105]. Hoang et al. [106] highlighted the importance of encapsulants in matrix formation and encapsulation efficiency, where the efficiency exhibited nonlinear behavior with respect to the IAT, increasing from 78.61% at 120 °C to 88.74% at 140 °C and decreasing to 76.22% at 180 °C; furthermore, the highest value was obtained at a feed flow rate of 7 mL/min. These results demonstrate that TAC protection depends on a combination of operating conditions and formulation.

#### 3.1.6. Antioxidant Capacity

Antioxidant capacity is an indicator that reflects the potential of TPC to neutralize reactive species, through the transfer of electrons or hydrogen [107]. In our study, the antioxidant capacity values of the açaí powder ranged from 3766.35 to 9975.55 µmol TE/g as measured by the ABTS assay, and from 3190.99 to 5754.02 µmol TE/g as measured by the DPPH assay. It was observed that antioxidant activity showed a trend concomitant with the TPC, which is consistent with the general trend described by Wilkowska et al. [108] noted that, in blueberry powders, formulations with higher TPC exhibited the greatest antioxidant activity, even surpassing those with higher TAC concentrations, which highlights the significant contribution of non-pigment phenolic compounds.

The values obtained using the ABTS method are consistent with those reported by Vargas et al. [35], who determined an activity of 11,745.53 µmol TE/g in açaí powders. However, the results were higher than those reported by Vargas et al. [84] who observed a range of 2601.84 to 4790.35 mg/100 g in açaí and cupuazú matrices. Regarding the DPPH assay, our results are comparable to those reported by Tonon et al. [31], who reported 1165.84 µmol TE/g. On the other hand Fernandes et al. [109] obtained DPPH values ranging from 3125.1 to 3406.0 μmol TE/g for *Psidium guajava* powders. In this context, Mrkìc et al. [110] suggest that partially oxidized polyphenols may, under certain processing conditions, enhance the effectiveness of free radical scavenging, which could explain the observations. Antioxidant activity (ABTS and DPPH) peaked at 120 °C and showed a significant decrease at 150 °C. The observed behavior suggests the existence of a critical temperature range; in this range, moderate IAT promote the formation of a protective crust, while excessive heat accelerates the thermo-oxidative degradation of the TPC before the biopolymer barrier has had a chance to form. Despite heat stress, the remarkable residual antioxidant capacity is attributed to the high initial concentration of TPC (ABTS r: 0.939; *p*: 0.001 and DPPH r: 0.963; *p*: 0.001) and to the protective efficacy of the GA and MD matrix.

**Figure 1:**
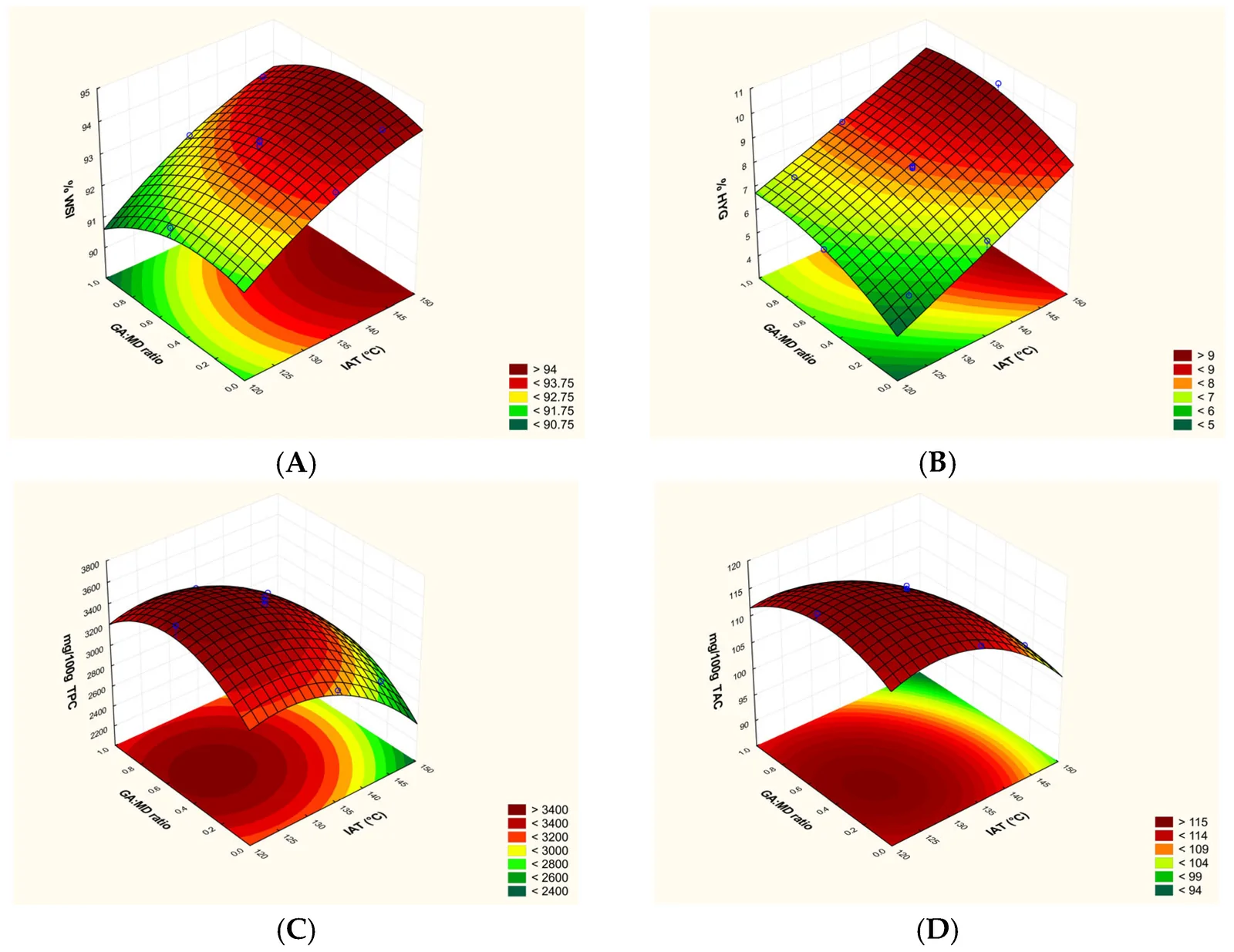
Three-dimensional response surface showing the effects of inlet air temperature (IAT) and the gum Arabic: maltodextrin (GA:MD) ratio; (**A**) water-solubility index (% WSI), (**B**) hygroscopicity (% HYG), (**C**) total phenolic content (mg GAE/100 g TPC), and (**D**) total anthocyanins content (mg C3G/100 g).

Figure 1 shows the response surfaces illustrating the effects of IAT and the GA:MD ratio on the response variables evaluated.

### 3.2. Experimental Validation of the Optimal Point of the Process

Model validation was performed simultaneously based on the desirability function (D). Table 4 shows the values assigned to the D function, where the responses were coded as undesirable (0), moderately desirable (0.5), and desirable (1), corresponding to the minimum, average, and maximum values, respectively. Likewise, importance and shape coefficients (S = T = 2) were established for all responses, applying a quadratic penalty to deviations from the desired objectives.

**Figure 2:**
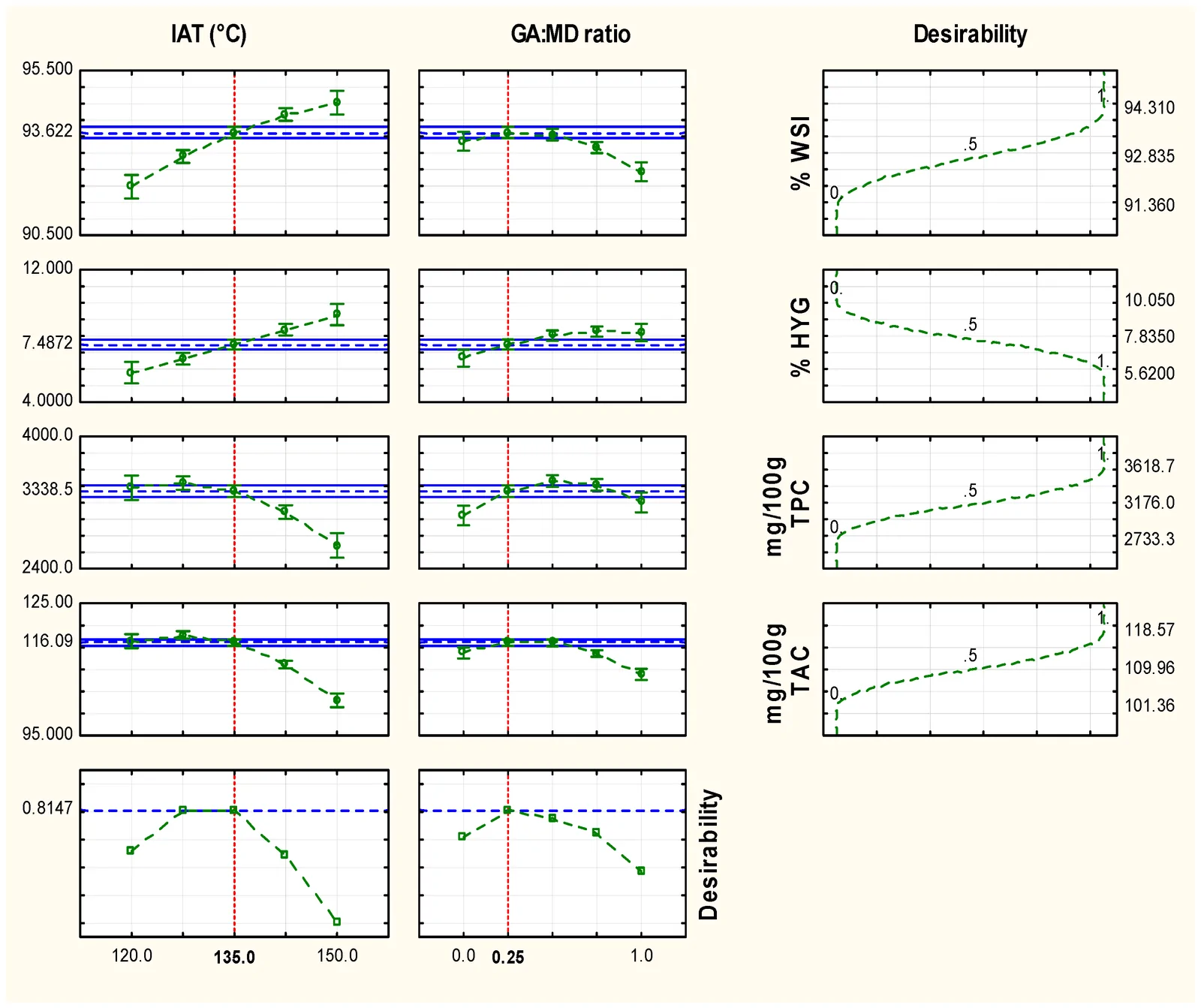
Desirability function (D) for simultaneous optimization of variables; effects of inlet air temperature (IAT) and gum Arabic: maltodextrin (GA:MD) ratio; water-solubility index (% WSI), hygroscopicity (% HYG), total phenols (mg GAE/100 g TPC), and total anthocyanins (mg C3G/100 g).

Optimization using the D-function (Figure 2) indicated that the most favorable conditions for spray-drying the açaí extract corresponded to an IAT of 135 °C and a GA:MD ratio of 25:75, parameters that achieve a “D” of 0.8147, which demonstrates a high degree of simultaneous fulfillment of the optimization objectives established for the responses. Desirability values between 0.80 and 1.00 are considered indicative of excellent optimization quality [111], indicating that, under these conditions, the model predicted values of 93.62% WSI, 7.49% HYG, 3338.50 mg GAE/100 g of TPC, and 116.09 mg C3G/100 g of TAC.

According to Table 5, the experimentally observed values fell within the 95% confidence interval of the predicted value, confirming the statistical robustness and predictive capability of the developed model. The GA:MD ratio of 25:75, identified as optimal in our study, is consistent with the literature reporting the superiority of MD-predominant formulations for the retention of bioactive compounds during spray drying. Da Silva Carvalho et al. [112] reported 96% retention of TAC (154.6 mg C3G/100 g) in juçara extract microparticles, where these retention levels were influenced by the encapsulants, which were present in a 25:75 GA:MD ratio. In this regard, the agreement between the predicted and observed values is also consistent with the results reported by Dang et al. [113] for response surface models used in the optimization of spray drying of acerola. The observed HYG and high WSI in our study are comparable to the optimized values reported by Tello et al. [7] for açaí powder, which had an HYG of 7.28 and 93.27% of WSI. Likewise, Decker et al. [57] reported a maximum CAT retention of 91.94% and a PY of 50.0% for grape pomace powder. In addition, Cid-Ortega and Guerrero-Beltrán [93] reported 3801.60 mg GAE/100 g of TPC using GA: MD. Therefore, the results of our study provide technical information for the processing of *Euterpe precatoria* using spray-drying, which makes the resulting product a candidate for use as a functional powder.

### 3.3. Morphological and Colloidal Properties of the Powder

**Figure 3:**
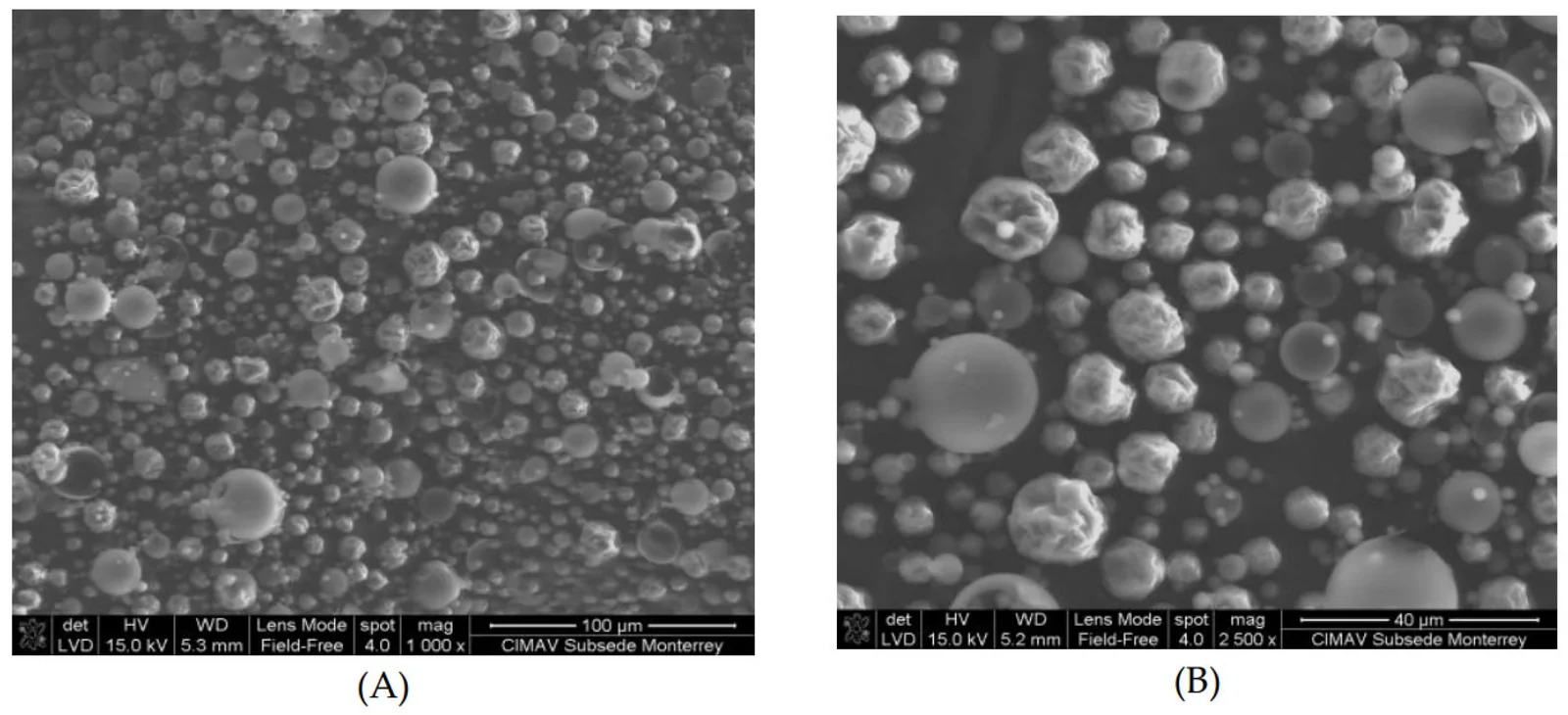
Scanning electron microscopy (SEM) micrographs of açaí (*Euterpe precatoria* Mart.) powder taken under optimal conditions at (**A**) 1000× and (**B**) 2500×.

SEM micrographs of açaí powder obtained under optimal spray-drying conditions showed predominantly spherical powders with smooth or slightly wrinkled surfaces, while some exhibited surface depressions attributable to the shrinkage of the encapsulating matrix during drying (Figure 3).

The predominance of spherical morphology is consistent with the findings reported by Tonon et al. [114] for açaí powders. These authors stated that the surface finish depends on both the composition of the biopolymers and the IAT that is associated with the rapid evaporation of water. In this context, the slight wrinkles observed in a fraction of the powder in this study can be interpreted as the result of differential contraction of the outer wall during the progressive loss of moisture in the drying chamber [76]. Similarly, da Silva Carvalho et al. [112] described spherical powders derived from juçara extracts, while Mazuco et al. [115] confirmed that formulations made with MD:GA produced juçara powders with a spherical geometry, in contrast to the irregular flakes obtained by freeze-drying.

The absence of obvious structural discontinuities, such as cracks or fractures, suggests adequate morphological integrity of the microparticle walls. Although it has been proposed that continuous structures may help to limit oxygen diffusion and promote the stability of sensitive compounds during storage [116], oxygen permeability was not determined in this study, which prevents us from establishing such a relationship. In this regard, Kalušević et al. [117] reported that powders obtained by spray-drying with MD and GA exhibit non-porous spherical structures, which were associated with greater TAC retention. However, because oxygen permeability was not measured in our study, it is not possible to establish a direct relationship between the two variables. Morphometric analysis indicated a powder size range between 2.10 and 24.22 μm, a result consistent with the mean value of 6.91 μm recorded by Vargas et al. [35] for açaí powders produced using MD. Similarly, de Oliveira Ribeiro et al. [118] reported açaí powders with a mean diameter of less than 10 μm, placing the values in the present study within the typical range for this species family in the genus *Euterpe*.

The ZP of the reconstituted açaí powder (*Euterpe precatoria*) ranged from −3.82 to −4.26 mV, while the PDI ranged from 0.368 to 0.398. The hydrodynamic diameter determined by DLS was 379.7 ± 30.1 nm (*n*: 3). The PDI values obtained indicate a relatively homogeneous size distribution, consistent with systems produced by spray drying. Generally, PDI values below 0.5 are considered acceptable for these systems, as they reflect moderate size dispersion and adequate microparticle uniformity [119]. These measurements describe the electrokinetic behavior and hydrodynamic size distribution of the dispersed structures present after the reconstitution of the powder in an aqueous medium. Low absolute ZP values indicate limited electrostatic stability of the reconstituted aqueous dispersions under the evaluated conditions. In colloidal systems, absolute values greater than approximately 25 mV are typically associated with intense electrostatic repulsion, whereas values close to 0 mV are associated with a greater tendency toward aggregation or flocculation [120,121]. In this context, the results obtained are comparable to those of Rigolon et al. [101] for açaí powder, where the dispersions were close to −5.0 mV. In contrast, Pereira et al. [122] reported −16.47 mV for *Origanum vulgare* oil powders. In this context, the influence of biopolymers as wall materials on electrokinetic properties has recently been described by da Silva Melo et al. [123], who observed that the incorporation of GA:MD can decrease the magnitude of the ZP due to the surface coating of the powder, thereby reducing the exposure of charged functional groups to the external environment. This phenomenon could partly explain the low values recorded in our study for açaí powder.

Consequently, the micrographs obtained by SEM provide qualitative information on the morphology of the microparticles in their dry state. However, the observed surface appearance does not allow for direct determination of porosity, oxygen permeability, moisture absorption, TAC location, or powder stability during storage; therefore, the contribution of the GA:MD matrix to the protection of TPC and TAC must be confirmed experimentally through the aforementioned tests.

## 4. Conclusions

RSM indicated that both the IAT and the GA:MD ratio significantly affected the evaluated responses (*p* < 0.05). For the responses included in the desirability function “D”, regression analysis confirmed the reliability of the quadratic models obtained, explaining more than 92% of the variability within the evaluated experimental range. The optimal conditions for spray-drying the açaí extract (*Euterpe precatoria* Mart.) were an IAT of 135 °C and a GA:MD ratio of 25:75. Experimental validation of the optimal conditions predicted by the models confirmed their predictive capability, achieving a “D” of 0.8147; under these conditions, the observed responses were 93.49% WSI, 7.42% HYG, 3291.22 mg GAE/100 g TPC, and 115.89 mg C3G/100 g TAC. These values fell within the reported confidence intervals; the relative errors between the observed and predicted values were less than 1.5%. Consequently, the powders exhibited relatively low HYG under the experimental conditions, exhibiting high solubility and high retention of TPC (up to 92.15%) and TAC (up to 84.60%), in addition to being predominantly spherical, with smooth or slightly wrinkled surfaces, and microparticle sizes ranging from 2.10 to 24.22 μm. This indicates that, under the conditions evaluated, favorable results were obtained in terms of the physicochemical properties and the retention of TPC and TAC in the powder, in accordance with the criteria established in the optimization process; this improves the characteristics of the final product. However, it is necessary to determine the Tg and the water activity, as well as to evaluate the powder’s stability during storage to ensure its functionality. Therefore, these results provide an experimental basis for the development of value-added products from Amazonian fruits.

## Figures and Tables

**Table 1:** Phenolic compounds in the pulp and hydroalcoholic extract of açaí.

Sample	TPC	TAC	ABTS	DPPH
Pulp	239.26 ± 5.11 ^b^	9.34 ± 1.09 ^b^	1151.24 ± 7.36 ^b^	917.59 ± 4.99 ^b^
Extract	3927.09 ± 6.80 ^a^	140.16 ± 1.55 ^a^	12,573.88 ± 16.78 ^a^	7763.28 ± 12.78 ^a^

Mean values ± standard deviation (*n* = 3). The means marked with different letters in the same column differ significantly from one another, according to Student’s *t*-test (*p* < 0.05). TPC: mg GAE/100 g; TAC: mg C3G/100 g; ABTS and DPPH: µmol TE/g.

**Table 2:** CCD and experimental results (PY, MC, WSI, HYG, TPC, TAC, ABTS, and DPPH) for spray drying of açaí.

Assay	Coded Values		Decoded Values				Response Variables					
	X1	X2	IAT	GA:MD Ratio	PY	MC	WSI	HYG	TPC	TAC	ABTS	DPPH
1	−1	−1	124.4	10:90	48.13	3.16	92.13	5.62	3174.06	115.04	9046.33	4705.02
2	−1	1	124.4	90:10	47.96	3.63	91.36	7.29	3295.37	113.06	9206.31	5310.63
3	1	−1	145.6	10:90	50.95	2.04	94.27	7.93	2820.43	107.75	5805.59	3971.79
4	1	1	145.6	90:10	49.31	2.45	93.77	9.31	3003.54	102.43	6661.23	4315.69
5	−1.415	0	120	50:50	49.88	3.24	92.19	6.36	3618.66	118.57	9975.55	5754.02
6	1.415	0	150	50:50	53.82	2.01	94.31	10.05	2733.32	101.36	3766.35	3190.99
7	0	−1.25	135	0:100	51.05	3.15	93.52	7.06	3089.94	114.32	7091.98	4661.76
8	0	1.25	135	100:0	48.74	4.06	92.43	8.15	3207.24	109.04	8048.03	5105.15
9	0	0	135	50:50	52.92	2.46	93.49	7.56	3420	116.31	9317.81	5483.66
10	0	0	135	50:50	52.21	2.52	93.63	8.17	3498.14	115.64	9405.07	5436.08
11	0	0	135	50:50	52.45	2.33	93.65	8.12	3535.09	116.06	9325.08	5440.4
12	0	0	135	50:50	52.53	2.44	93.65	8.08	3390.21	115.25	9322.65	5462.03
13	0	0	135	50:50	52.98	2.55	93.33	8.1	3460.12	116.85	9317.62	5464.19

X1: inlet air temperature (IAT °C); X2: biopolymer ratio (GA:MD); PY: %; WSI: %; HYG: %; TPC: mg GAE/100 g; TAC: mg C3G/100 g; ABTS and DPPH: µmol TE/g radical scavenging activity.

**Table 3:** ANOVA of the quadratic response surface model for the parameters (PY, MC, WSI, HYG, TPC, TAC, ABTS, and DPPH) of the spray-drying process of açaí.

Factor	Sum of Squares	Degree of Freedom	Mean Square	F-Value	*p*-Value
% PY					
X_1_	11.86	1	11.86	111.75	0.00
$X_{1}^{2}$	3.63	1	3.63	34.20	0.00
X_2_	3.10	1	3.10	29.17	0.01
$X_{2}^{2}$	25.74	1	25.74	242.40	0.00
X_1_X_2_	0.54	1	0.54	5.09	0.09
Lack of fit	4.01	3	1.34	12.61	0.02
Pure error	0.42	4	0.11		
Total, SS	48.17	12			
R^2^	0.9078				
% MC					
X_1_	2.04	1	2.04	281.31	0.00
$X_{1}^{2}$	0.00	1	0.00	0.65	0.47
X_2_	0.57	1	0.57	78.80	0.00
$X_{2}^{2}$	1.55	1	1.55	213.69	0.00
X_1_X_2_	0.00	1	0.00	0.12	0.74
Lack of fit	0.43	3	0.14	19.82	0.01
Pure error	0.03	4	0.01		
Total, SS	4.64	12			
R^2^	0.9009				
% WSI					
X_1_	7.12	1	7.12	363.31	0.00
$X_{1}^{2}$	0.24	1	0.24	12.38	0.02
X_2_	0.97	1	0.97	49.62	0.00
$X_{2}^{2}$	0.90	1	0.90	45.71	0.00
X_1_X_2_	0.02	1	0.02	0.93	0.39
Lack of fit	0.36	3	0.12	6.21	0.06
Pure error	0.08	4	0.02		
Total, SS	9.64	12			
R^2^	0.9540				
% HYG					
X_1_	11.40	1	11.40	180.11	0.00
$X_{1}^{2}$	0.00	1	0.00	0.07	0.80
X_2_	2.73	1	2.73	43.18	0.00
$X_{2}^{2}$	0.70	1	0.70	11.04	0.03
X_1_X_2_	0.02	1	0.02	0.33	0.60
Lack of fit	0.45	3	0.15	2.38	0.21
Pure error	0.25	4	0.06		
Total, SS	15.57	12			
R^2^	0.9548				
mg GAE/100 g TPC					
X_1_	450,162.23	1	450,162.23	132.78	0.00
$X_{1}^{2}$	165,228.80	1	165,228.80	48.74	0.00
X_2_	28,553.21	1	28,553.21	8.42	0.04
$X_{2}^{2}$	233,600.17	1	233,600.17	68.90	0.00
X_1_X_2_	954.81	1	954.81	0.28	0.62
Lack of fit	50,936.50	3	16,978.83	5.01	0.08
Pure error	13,561.29	4	3390.32		
Total, SS	918,972.14	12			
R^2^	0.9298				
mg C3G/100 g TAC					
X_1_	223.24	1	223.24	590.64	0.00
$X_{1}^{2}$	71.35	1	71.35	188.76	0.00
X_2_	27.12	1	27.12	71.74	0.00
$X_{2}^{2}$	45.40	1	45.40	120.10	0.00
X_1_X_2_	2.79	1	2.79	7.38	0.05
Lack of fit	5.99	3	2.00	5.29	0.07
Pure error	1.51	4	0.38		
Total, SS	370.44	12			
R^2^	0.9797				
µmol TE/g ABTS					
X_1_	26,527,915.00	1	26,527,915.00	18,540.33	0.00
$X_{1}^{2}$	7,996,708.54	1	7,996,708.54	5588.89	0.00
X_2_	685,911.17	1	685,911.17	479.38	0.00
$X_{2}^{2}$	3,683,909.68	1	3,683,909.68	2574.68	0.00
X_1_X_2_	120,985.71	1	120,985.71	84.56	0.00
Lack of fit	1,999,833.63	3	666,611.21	465.89	0.00
Pure error	5723.29	4	1430.82		
Total, SS	40,360,083.97	12			
R^2^	0.9503				
µmol TE/g DPPH					
X_1_	3,582,404.16	1	3,582,404.16	9549.33	0.00
$X_{1}^{2}$	1,744,809.41	1	1,744,809.41	4651.00	0.00
X_2_	317,369.34	1	317,369.34	845.99	0.00
$X_{2}^{2}$	700,076.50	1	700,076.50	1866.14	0.00
X_1_X_2_	17,123.03	1	17,123.03	45.64	0.00
Lack of fit	455,986.37	3	151,995.46	405.16	0.00
Pure error	1500.59	4	375.15		
Total, SS	6,684,974.72	12			
R^2^	0.9316				

X1: inlet air temperature (IAT °C); X2: biopolymer ratio (GA:MD); PY: %; WSI: %; HYG: %; TPC: mg GAE/100 g; TAC: mg C3G/100 g; ABTS and DPPH: µmol TE/g radical scavenging activity.

**Table 4:** Parameters used to optimize the process response variables.

Response	Low	Medium	High	S (*)	T (*)
% WSI	91.36 (0.0)	92.84 (0.5)	94.31 (1.0)	2	2
% HYG	5.62 (1.0)	7.84 (0.5)	10.05 (0.0)	2	2
mg GAE/100 g TPC	2733.32 (0.0)	3175.99 (0.5)	3618.66 (1.0)	2	2
mg C3G/100 g TAC	101.36 (0.0)	109.96 (0.5)	118.57 (1.0)	2	2

*: S and T, exponents of the desirability function.

**Table 5:** Validation of the optimization using desirability function (D).

Response	Observed Values	Predictive Values	95% CI for Mean Predicted Response	95% PI for a New Observation	% Relative Deviation
% WSI	93.49 ± 1.11	93.62	93.45–93.79	93.20–94.05	0.14%
% HYG	7.42 ± 0.38	7.49	7.18–7.79	6.72–8.25	0.94%
mg GAE/100 g TPC	3291.21 ± 0.46	3338.50	3267.57–3409.46	3161.97–3515.06	1.44%
mg C3G/100 g TAC	115.89 ± 0.79	116.09	115.34–116.84	114.22–117.95	0.17%

Mean value ± standard error, based on three replicates (*n* = 3).

## Data Availability

The data presented in this study are available upon request to the corresponding author.

## Abbreviations

The following abbreviations are used in this manuscript:

MD	maltodextrin
GA	gum Arabic
IAT	inlet air temperature
Tg	glass transition temperature
PY	process yield
MC	residual moisture content
WSI	water-solubility index
HYG	hygroscopicity
TAC	total anthocyanin
TPC	total phenolic
GAE	gallic acid equivalents
C3G	cyanidin-3-glucoside
ABTS	2,2′-Azino-bis (3-ethylbenzothiazoline-6-sulfonic acid)
DPPH	2,2-Diphenyl-1-picrylhydrazyl
CCD	central composite design
ANOVA	analysis of variance
R^2^	coefficient of determination
RSM	response surface methodology
ZP	Zeta potential
